# Reanalysis of 2 metritis studies demonstrates different patterns of postpartum uterine infection for primiparous versus multiparous cows

**DOI:** 10.3168/jdsc.2024-0679

**Published:** 2025-01-16

**Authors:** J.C.C. Silva, M.C. Lucy

**Affiliations:** Division of Animal Sciences, University of Missouri, Columbia, MO 65211

## Abstract

Metritis typically has a greater incidence in primiparous compared with multiparous cows. In separate studies with similar design, we noted that primiparous and multiparous cows responded differently to a model developed to induce metritis via the intrauterine infusion of a bacterial challenge of *Fusobacterium necrophorum*, *Trueperella pyogenes*, and *Escherichia coli*. To understand the difference between primiparous and multiparous cows, we reanalyzed the relative abundance of bacteria genera within the vaginal microbiome during the first 2 wk postpartum from the 2 previous studies for primiparous and multiparous cows. We conducted a first reanalysis of primiparous and multiparous cows that received an identical challenge dose (10^6^ cfu of each pathogen) and a second reanalysis that compared all primiparous and multiparous cows that were or were not diagnosed with metritis regardless of challenge dose (0, 10^3^, 10^6^, or 10^9^ cfu per pathogen). The challenge model resulted in clinical metritis in both primiparous and multiparous cows, although some control cows (0 dose) developed metritis and, conversely, some bacterial challenge cows failed to develop metritis. Importantly, cows that contracted metritis had increased and sustained relative abundance of key metritis pathogens including *Fusobacterium*, *Porphyromonas*, *Helcococcus*, and *Trueperella* after calving regardless of parity. We unexpectedly found, however, that primiparous cows that did not develop metritis had a different bacterial profile (based on 16S ribosomal gene sequencing as well as bacterial culture) compared with multiparous cows that did not develop metritis. In primiparous nonmetritis cows, the relative abundance of the genera *Fusobacterium*, *Porphyromonas*, *Helcococcus*, and *Trueperella* was almost identical to primiparous metritis cows during the first week postpartum, but the relative abundances in nonmetritis cows decreased rapidly thereafter. The relative abundance of the same genera in nonmetritis multiparous cows did not increase or increased to a lesser extent postpartum. The different patterns of infection for nonmetritis primiparous (initial increase in relative abundance [wk 1] followed by a decrease [wk 2]) compared with nonmetritis multiparous cows (stable and low-level relative abundance for 2 wk postpartum) was found when the analysis included only challenge cows (10^6^ cfu dose) or all cows regardless of dose. We found different patterns of infection for primiparous compared with multiparous cows. This observation may explain lesser incidence of metritis in multiparous compared with primiparous cows.

## Highlights

• Cows had an increase in metritis-causing pathogens during the first 2 weeks postpartum.
• Primiparous cows that remained healthy had increased metritis-causing pathogens in week 1.
• Multiparous cows that remained healthy had a different pattern than primiparous cows.
• We found different patterns of infection for primiparous compared with multiparous cows.

## Graphical Abstract

**Figure:**
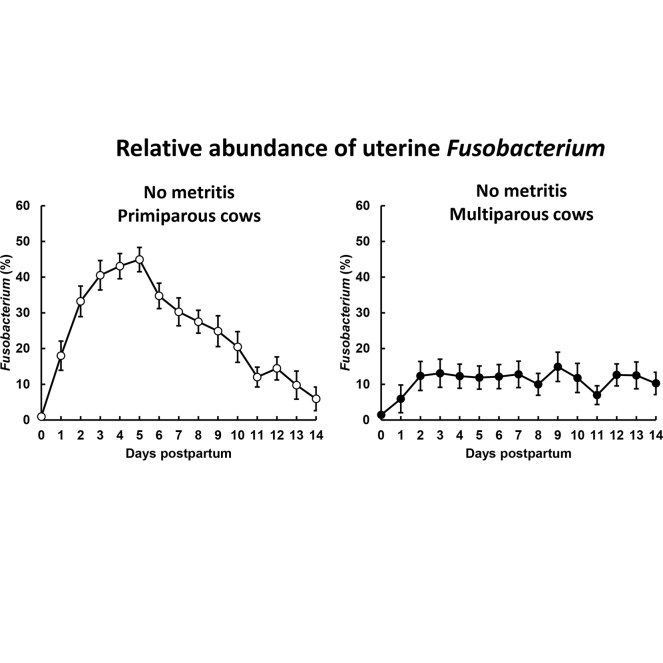
**Summary:** Metritis typically has a greater incidence in primiparous compared with multiparous cows. To identify biological mechanisms that underlie the observed parity difference in metritis incidence, we reanalyzed the relative abundance of bacterial genera from 2 previous studies that tested a metritis challenge model in primiparous and multiparous cows. We unexpectedly found that primiparous cows that failed to develop metritis (remained healthy) had a different bacterial profile (relative abundance) than multiparous cows that failed to develop metritis following challenge. Specifically, healthy primiparous cows had an initial increase (week 1 postpartum) followed by a decrease (week 2 postpartum) in the relative abundance of metritis pathogens, suggesting the establishment followed by resolution of infection. For healthy multiparous cows, relative abundance remained low following the challenge. In this reanalysis of 2 independent studies, we found different patterns of infection for primiparous compared with multiparous cows.

Uterine disease during the first week postpartum (metritis) affects 10% to 25% of cows after calving (Bromfield et al., 2015; Galvão et al., 2019; Sheldon et al., 2020). Metritis is an important disease during the transition period because cows that develop metritis require treatment, have reduced milk production, and have reduced fertility later postpartum (LeBlanc, 2023). Dairy cows suffer from a variety of illnesses postpartum (mastitis, ketosis, lameness, and so on) all of which have increased incidence as the cow ages (i.e., progresses through successive parities; Lean et al., 2023a,b). Based on a retrospective meta-analysis involving more than 28,000 cows in Australia, Canada, and the United States, metritis was the only disease that had greater incidence in first parity (primiparous) cows compared with all other second or greater parity cows (multiparous; Lean et al., 2023b). In an effort to establish a metritis challenge model, Silva et al. propagated well-characterized metritis-causing strains of *Fusobacterium necrophorum*, *Trueperella pyogenes*, and *Escherichia coli* for intrauterine infusion into Holstein cows immediately after calving as described previously for primiparous (Silva et al., 2023a) and multiparous cows (Silva et al., 2023b). Given the reported differences for primiparous versus multiparous cows for metritis incidence (Lean et al., 2023b) and their different responses to a bacterial challenge (Silva et al., 2023a,b), we combined the data from the original Silva et al. studies to test the effect of parity on the uterine microbiome of metritis and nonmetritis cows after calving. The hypothesis was that the primiparous and multiparous cows would differ with respect to the relative abundance of metritis pathogens following a bacterial challenge and that these differences would explain the greater incidence of metritis in primiparous compared with multiparous cows as reported in the retrospective analysis of Lean et al. (2023b).

Both studies were reviewed and approved by the Cornell University Institutional Animal Care and Use Committee (protocol number 2016–0040). Across both challenge models (Silva et al., 2023a,b), cows had either control saline (n = 12 primiparous and n = 11 multiparous), infused with 10^3^ cfu of bacterial cocktail (n = 12 primiparous), infused with 10^6^ cfu of bacterial cocktail (n = 11 primiparous and n = 12 multiparous), or infused with 10^9^ cfu of bacterial cocktail (n = 12 multiparous; Table 1). The multiparous cows were parity 2 (n = 16), parity 3 (n = 17), parity 4 (n = 1), or parity 5 (n = 1). The bacterial cocktail included equal numbers (10^3^, 10^6^, or 10^9^ cfu per pathogen) of *F. necrophorum*, *T. pyogenes*, or *E. coli*. The investigators removed potential confounding effects of retained placenta on metritis (Sheldon et al., 2019) by only using cows that expelled the placenta. Also, any cow that had any factor that would predispose her to metritis including dystocia was not enrolled or was removed from the study. Clinical disease onset following the challenge was within the first 14 d after calving (Silva et al., 2023a,b). In this study, we reanalyzed the data for the 16S rDNA sequencing (metagenomics) and bacteriology (culture) of the vaginal swab samples that were collected from d 0 to 14 postpartum from the primiparous (Silva et al., 2023a) and multiparous (Silva et al., 2023b) cows. The swab samples were subjected to DNA extraction and 16S rDNA sequencing of the V4 hypervariable region. The metagenomic sequencing generated amplicon sequence variants (**ASV**) that were collapsed to the genus level. The relative abundance of genus was calculated within cow using the equation: relative abundance = number of ASV counts of each microorganism/sum of all counts within the individual cow, expressed as a percentage. Bacterial culture of *F. necrophorum*, *T. pyogenes*, or *E. coli* was also conducted as described in the original publications. The relative abundance of individual ASV or log_10_ cfu over time (i.e., first 14 d postpartum) was analyzed. The PROC UNIVARIATE procedure of SAS version 9.4 (SAS Institute Inc., Cary, NC) was used to assess normality of the original data. Skewness and kurtosis values of −2 to 2 were considered acceptable for the normality test (George and Mallery, 2010). The relative abundance (based on ASV) of *Fusobacterium* was normally distributed. Data for the relative abundance of all other bacterial genera were nonnormal, so the relative abundance was log_10_ transformed to establish normality before the statistical analysis using PROC MIXED of SAS. Culture data (log_10_ cfu) were normally distributed. Separate statistical models were fitted for metritis (vaginal discharge score = 3 [fetid, watery, red-brownish uterine discharge] according to the classification system of Sheldon et al., 2006) and nonmetritis cows (vaginal discharge score of 0, 1, or 2) using the PROC MIXED procedure of SAS and included the fixed effects of parity, day postpartum, and parity × days postpartum interaction. In the first set of analyses, we included only cows that were treated with the 10^6^ challenge dose (this dose was applied across both of the original studies). A random effect of cow within parity was included. A second analysis included all groups (doses of 0, 10^3^, 10^6^, or 10^9^ cfu per pathogen; Table 1) with separate analyses for metritis or nonmetritis cows using the fixed effects of parity, day postpartum, and parity × day postpartum interaction. A random effect of cow within parity was included. The heterogeneous compound symmetry covariance structure was used for all models. Significance was defined as a *P*-value ≤0.05 and tendency was defined as a *P*-value between 0.05 and 0.10.

**Table 1:** Number of individual primiparous and multiparous cows that contracted metritis (Met) or remained healthy (He) following an intrauterine challenge dose (0, 10^3^, 10^6^, or 10^9^ cfu per pathogen) of *Fusobacterium necrophorum*, *Trueperella pyogenes*, and *Escherichia coli* infused within 24 h after parturition^1^

Group	Dose											
	0 (control)			10^3^ cfu			10^6^ cfu			10^9^ cfu		
	Met	He	Total	Met	He	Total	Met	He	Total	Met	He	Total
Primiparous	4	8	12	5	7	12	7	4	11	—	—	—
Multiparous	1	10	11	—	—	—	10	2	12	3	9	12
Total	5	18	23	5	7	12	17	6	23	3	9	12

1 Data are summarized from the publications of Silva et al. (2023a,b).

For control (saline-infused) across the 2 studies, 4 out of 12 (33%) primiparous cows and 1 out of 11 (9%) multiparous cows developed metritis (Table 1). The numerically greater percentage of control cows with metritis is consistent with larger datasets that show a greater incidence of metritis in primiparous compared with multiparous cows (Lean et al., 2023b). Across all bacterial challenge doses, 12 out of 23 (52%) of primiparous cows and 13 out of 24 (54%) multiparous cows had metritis. Within the 10^6^ cfu challenge group (the only identical dose infused across both studies) 7 out of 11 (64%) and 10 out of 12 (83%) primiparous and multiparous cows had metritis (respectively). When the data for both challenge models were combined, the 10^6^ dose increased the percentage of metritis cows compared with saline-infused control (17 out of 23 [74%] for 10^6^ dose compared with 5 out of 23 [22%] for control; χ^2^ = 10.5; *P* = 0.001). The onset of metritis diagnosis for primiparous and multiparous cows was similar (6.6 ± 0.7 d and 7.2 ± 0.7 d postpartum; *P* = 0.56) with a median of 6 and 7.5 d and interquartile range of 3.5 and 4 d (primiparous and multiparous cows, respectively).

We reanalyzed the data from the 10^6^ challenge dose because both original studies included this dose. For cows that developed metritis (Figure 1A) there was a sustained increase in the relative abundance for the genus *Fusobacterium* during the first 14 d postpartum (effect of day; *P* < 0.001). There was a parity × day interaction (*P* < 0.001) explained by a greater relative abundance for *Fusobacterium* for primiparous metritis cows compared with multiparous metritis cows during wk 1 (Figure 1A). The overall pattern for relative abundance of *Fusobacterium* differed for primiparous and multiparous nonmetritis cows (Figure 1B). For primiparous nonmetritis cows, the relative abundance of *Fusobacterium* increased to almost 50% within 3 d in a manner that was almost identical to that seen in primiparous metritis cows (Figure 1A). Thereafter, the relative abundance of *Fusobacterium* decreased in nonmetritis primiparous cows reaching approximately 10% relative abundance by 14 d (Figure 1B). In contrast, the relative abundance of *Fusobacterium* in nonmetritis multiparous cows never exceeded 10% across all sample days (Figure 1B). Bacterial culture of *F. necrophorum* from challenged cows gave similar results with a greater (*P* = 0.021) number of cfu for primiparous metritis compared with multiparous metritis cows (Figure 1C) and a tendency (*P* = 0.073) for a greater number of cfu for nonmetritis primiparous compared with nonmetritis multiparous cows (Figure 1D).

**Figure 1:**
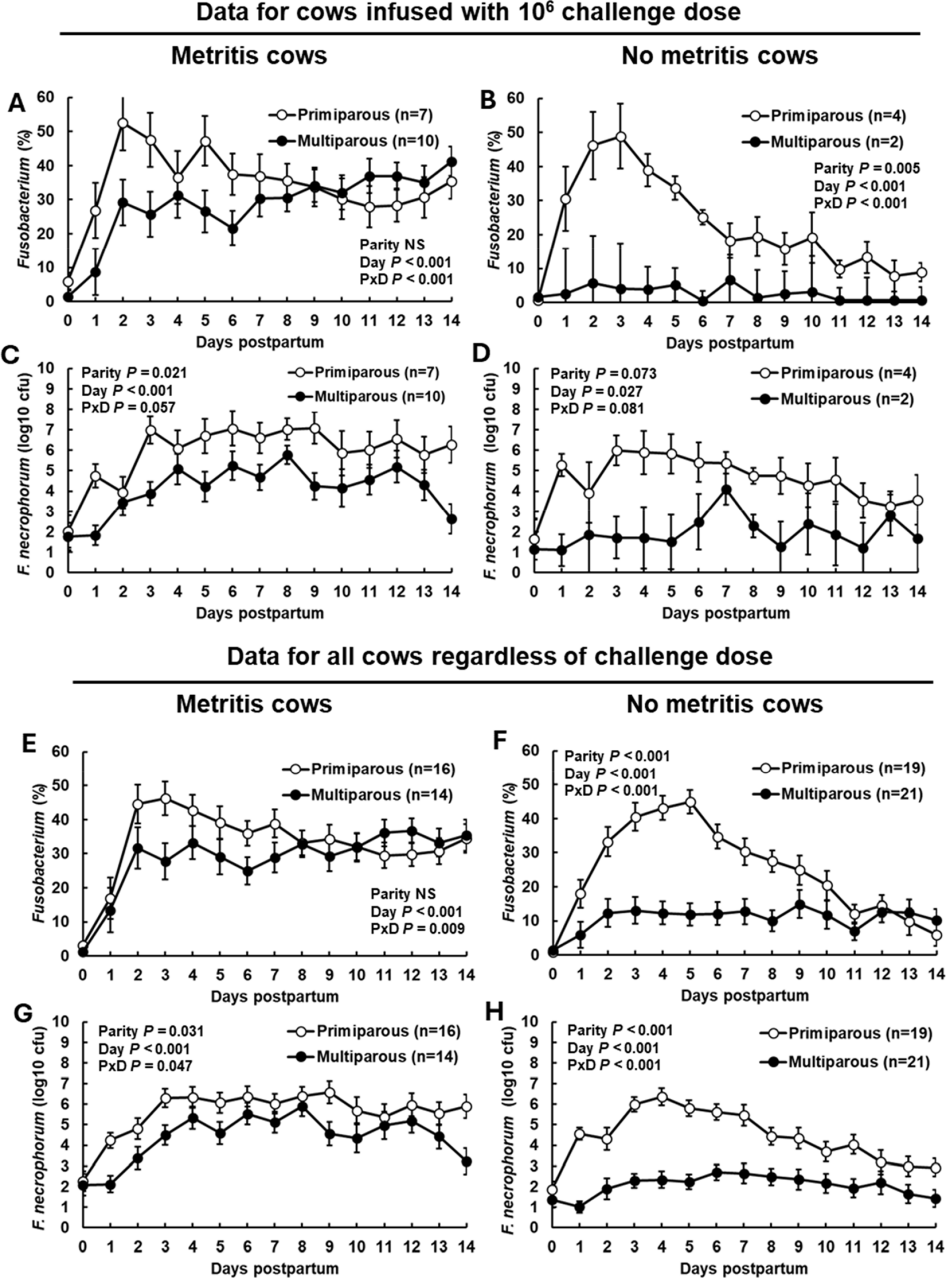
Relative abundance of the genus *Fusobacterium* (16S rDNA sequencing; A, B, E, and F) and number of cfu for bacterial culture of *Fusobacteriumnecrophorum* (C, D, G, and H) from vaginal content during the first 14 d postpartum for cows that were either primiparous or multiparous and either developed metritis (left panels A, C, E, and G) or remained healthy (right panels B, D, F, and H). Results depicted in the top 4 panels (A, B, C, and D) are for cows infused with a challenge dose containing 10^6^ cfu of *F. necrophorum*, *Trueperella pyogenes*, or *Escherichia coli* within 24 h after parturition (see Table 1). Results for the bottom panels (E, F, G, and H) are for all cows that developed metritis or remained healthy, regardless of their challenge dose. Results are presented as LSM ± SEM (bar). PxD = parity × day interaction. NS = not significant.

The challenge dose comparison included a relatively small number of nonmetritis cows (Table 1). We performed a subsequent analysis of all cows in the 2 studies that included all groups (doses of 0, 10^3^, 10^6^, or 10^9^ cfu per pathogen) and separated cows based on parity (primiparous or multiparous) and disease status (metritis or nonmetritis). This reanalysis was more robust given the large number of cows included in the collective experiments (n = 70) and the homogeneity of the sample size within the groups (primiparous metritis [n = 16], multiparous metritis [n = 14], primiparous nonmetritis [n = 19], and multiparous nonmetritis [n = 21]). As expected and similar to the 10^6^ challenge dose comparison (Figure 1A), cows with metritis had an increase in the relative abundance of *Fusobacterium* within 3 d postpartum and this increase persisted until 14 d postpartum (Figure 1E). As with the 10^6^ challenge dose (Figure 1A), there was a parity × day interaction (*P* < 0.009) because the primiparous cows had a greater relative abundance of *Fusobacterium* during the first 7 d postpartum when compared with the multiparous cows (Figure 1E). For nonmetritis cows (Figure 1F), the relative abundance increased in primiparous cows to almost 50% within 5 d postpartum before steadily decreasing to d 14 to approximately 10% relative abundance. Nonmetritis multiparous cows were different from primiparous cows in that the relative abundance of *Fusobacterium* increased to approximately 10% and remained relatively constant (Figure 1F; parity × day, *P* < 0.001). The culture data supported the data for relative abundance. The cfu for *F. necrophorum* increased in metritis cows with a parity × day interaction (*P* = 0.047) apparently caused by greater cfu in primiparous cows compared with multiparous cows within the first 7 d postpartum (Figure 1G). The *F. necrophorum* culture data for the nonmetritis cows (Figure 1H) demonstrated an almost identical pattern to that of the relative abundance of the genera *Fusobacterium* (Figure 1F). Specifically, the log_10_ cfu for *F. necrophorum* increased in nonmetritis primiparous cows within the first week postpartum and subsequently decreased. This pattern was not found within the nonmetritis multiparous cows where *F. necrophorum* remained relatively constant during the first 14 d postpartum (parity × day; *P* < 0.001; Figure 1H).

We reanalyzed the relative abundance of the most prevalent bacterial genera that we detected by 16S rDNA sequencing to determine if other genera demonstrated an effect of parity like what we observed for *Fusobacterium.* The additional genera were *Ureaplasma*, *Bacteroides*, *Streptococcus*, *Porphyromonas*, *Sneathia*, *Mycoplasma*, *Gallibacterium*, *Ruminococcus*, *Trueperella*, *Helcococcus*, and *Escherichia*. Among these, *Porphyromonas* had a pattern of relative abundance that was similar to *Fusobacterium* in that there was an increase in relative abundance in metritis cows (Figure 2A; effect of day; *P* < 0.001) and a distinct parity × day interaction (*P* < 0.001) for nonmetritis cows (Figure 2B), where primiparous cows compared with multiparous cows had a greater relative abundance during the first week postpartum. *Helcococcus* demonstrated an effect of day (*P* < 0.001) and a tendency for an effect of parity (*P* = 0.092) in metritis cows (Figure 2C) and an effect of parity × day in nonmetritis cows (Figure 2D). For *Helcococcu*s, the effect of parity × day (*P* < 0.001) in nonmetritis cows was explained by greater relative abundance in primiparous cows compared with multiparous cows during the first week after calving (Figure 2D). *Trueperella*, a bacterial genus also associated with metritis (Figure 2E), had a pattern of relative abundance that was similar to *Fusobacterium*, *Porphyromonas*, and *Helcococcus* in the nonmetritis primiparous cows (peaking in wk 1 and then decreasing) and this pattern was not found in the nonmetritis multiparous cows that had a sustained increase in *Trueperella* (Figure 2F; parity × day, *P* < 0.004). None of the other abundant genera demonstrated patterns of relative abundance that were similar to what we observed in *Fusobacterium*, *Porphyromonas*, *Helcococcus*, or *Trueperella*.

**Figure 2:**
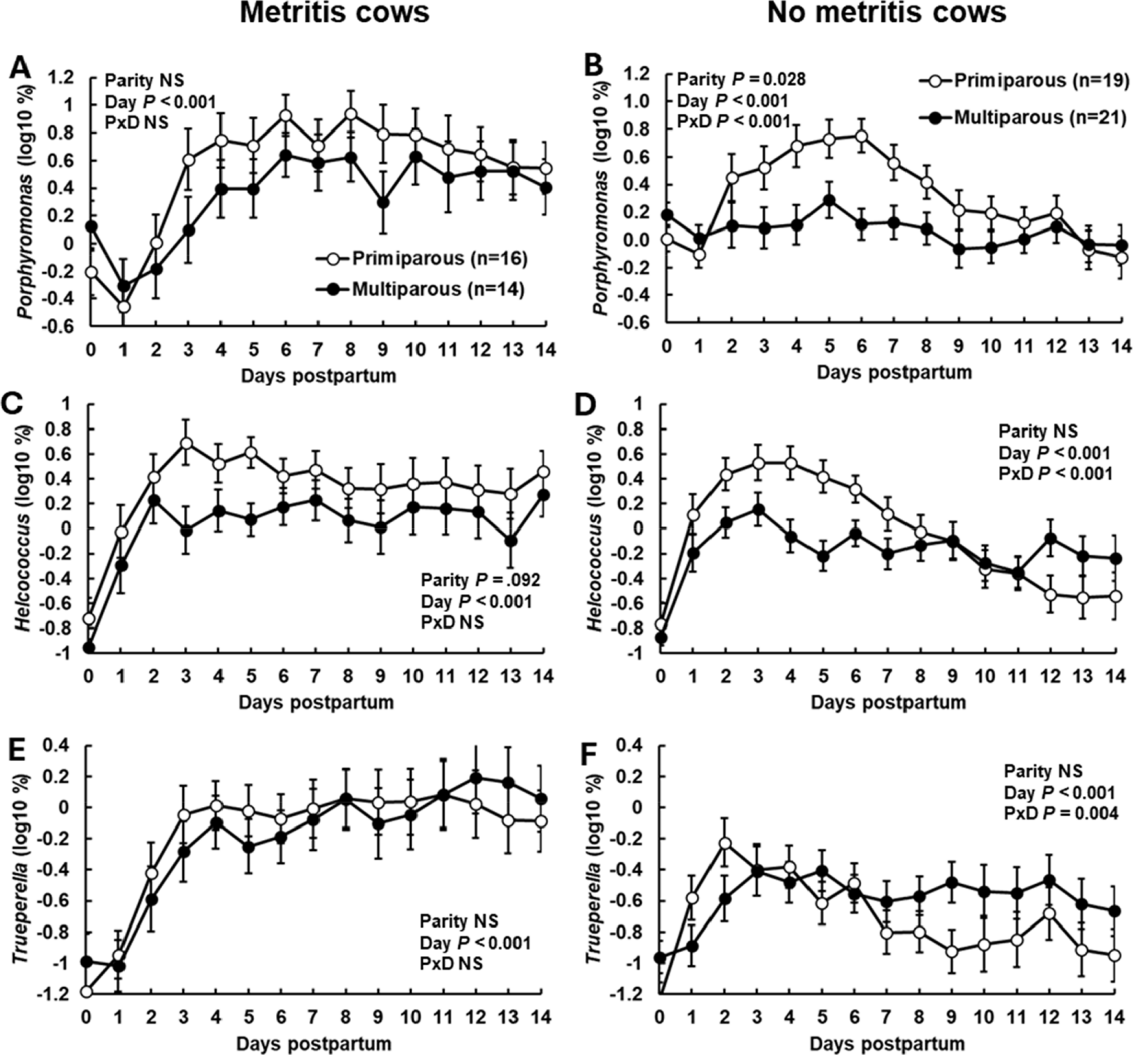
Relative abundance (based on 16S rDNA sequencing) of the genera *Porphyromonas* (A and B), *Helcococcus* (C and D), and *Trueperella* (E and F) from vaginal content during the first 14 d postpartum for cows that were either primiparous or multiparous and diagnosed with metritis (A, C, and E) or remained healthy (no metritis; B, D, and F). All cows, regardless of their challenge dose (0, 10^3^, 10^6^, or 10^9^ cfu per pathogen; infused within 24 h after parturition [d 0]), were included in the analysis. Results are presented as LSM ± SEM (bar). PxD = parity × day interaction. NS = not significant.

This reanalysis of existing datasets provides insight into the greater incidence of metritis in primiparous compared with multiparous dairy cows. Specifically, we find that important genera of pathogenic bacteria have different patterns of uterine infection in primiparous versus multiparous cows (based on vaginal microbiome). In their review article, Sheldon et al. (2019) described independent mechanisms for the pathogenesis of uterine disease, namely tolerance and resistance (Sheldon et al., 2019). Tolerance involves the mechanisms through which the tissue itself limits the damage caused by the pathogen. Resistance depends on the innate and adaptive immune systems. Resistance to metritis is typically ascribed to the cells of the innate immune system (mainly phagocytic cells including neutrophils as well as macrophages and dendritic cells) with lesser importance of the adaptive immune system (Bromfield et al., 2015; Sheldon et al., 2019). Although this study does not specifically address tolerance or resistance, we propose the possibility that the mechanisms of tolerance and resistance may differ for primiparous and multiparous cows and these different mechanisms may explain the very different patterns of infection.

The general pattern that we observed was largely similar for cows with metritis (Figures 1A, 1C, 1E, 1G, 2A, 2C, and 2E) where there was a sustained increase in relative abundance or cfu of metritis pathogens in cows with metritis regardless of parity. Parity, however, did affect the relative abundance or cfu of metritis pathogens in cows that did not develop metritis (Figures 1B, 1D, 1F, 1H, 2B, 2D, and 2F). Nonmetritis primiparous cows had a large increase in pathogen abundance (*Fusobacterium* [Figures 1B, 1D, 1F, and 1H], *Porphyromonas* [Figure 2B], *Helcococcus* [Figure 2D], and *Trueperella* [Figure 2F]) during wk 1 that was followed by a steady decline in pathogen abundance. This pattern was generally not observed in nonmetritis multiparous cows where the pathogen abundance remained relatively low (*Fusobacterium* and *Porphyromonas*) or increased but remained at a low and sustained level during the sampling period (*Helcococcus* and *Trueperella*).

Tolerance to pathogens mainly involves physical barriers and secreted products of the endometrial epithelium (Sheldon et al., 2019). The incidence of dystocia is greater in primiparous compared with multiparous cows (Lean et al., 2023b) and this may potentially explain some of the results that we observed. The uterine architecture may experience greater damage in primiparous cows at parturition and this greater damage may enable a more robust infection in primiparous when compared with multiparous cows. We do not believe that differences in the prepartum microbiome explain the different infection patterns because we did not find differences in the pregnancy microbiome for pregnant heifers compared with older cows (Moraes et al., 2024).

Resistance involves both the innate and adaptive immune systems with most of the metritis research focused on the innate immune system that is clearly the first line of defense to infection (Bromfield et al., 2015). Based on the infection patterns that we observed, it appeared that the innate immune system was highly active in the primiparous cows with their ability to rapidly clear the pathogen load that was established during wk 1 to remain healthy (see Figures 1B, 1D, 1F, 1H, 2B, 2D, and 2F). Low abundance in nonmetritis multiparous cows does not appear to involve “selection bias” (i.e., cows that do not develop metritis are those that were never exposed to pathogens) because the results of the challenge studies demonstrated that there were multiparous cows inoculated with the 10^6^ dose that failed to have an increase in *Fusobacterium* (Figure 1B).

The adaptive immune system is generally not thought to provide significant protection against metritis (Sheldon et al., 2019). Nonetheless, we propose based on these observations that first calving and the exposure to specific uterine pathogens at first calving may provide some protection at second calving through a mechanism that involves immunological memory. This memory most likely involves the adaptive immune system (Laidlaw et al., 2016) or possibly a trained innate immune system (Ochando et al., 2023; Bhargavi and Subbian, 2024). Most studies do not support a major role of adaptive immunity in the development of metritis (Bromfield et al., 2018) but there is evidence for reduced metritis incidence following vaccination (Machado et al., 2014; Machado and Silva, 2020). This adaptive mechanism did not appear to apply equally to all genera because we noted a smaller or nonexistent effect of parity for other pathogenic and nonpathogenic genera that we examined. Different species of bacteria have evolved with different capacity for evading host adaptive and innate immune systems (Hornef et al., 2002). This could explain the results that we observed if some species (but not all species) avoid an adaptive immune response. A second possibility is that there is trained immunity within the innate immune system of multiparous cows. The term “trained immunity” is used to describe epigenetic programming of the cells of the innate immune system that enable a more robust response upon a second antigen challenge (Ochando et al., 2023; Bhargavi and Subbian, 2024).

We acknowledge limitations of this reanalysis. First, the 2 studies were not conducted in a contemporaneous manner. The potential for confounding effects introduced in the separate studies needs to be considered within the context of the very large differences that were observed for nonmetritis primiparous and multiparous cows. Second, the original studies were done with vaginal sampling (Bicalho et al., 2017). We are assuming that the discharge of uterine lochia into the vagina gives an accurate representation of the uterine microbiome. Third, most of the multiparous cows in this study were parity 2 or 3. The data of Lean et al. (2023b) show greater metritis incidence in first-parity compared with multiparous cows. There are data from other studies that show as U-shaped response curve where older cows (third parity or greater) have an incidence of metritis that equals that found in first-parity cows (Bruun et al., 2002). Finally, we have combined cows that were control and treated with different doses within the different parity groups according to whether they were ultimately diagnosed as metritis or healthy. This analysis assumes that the dose did not affect the progression of the disease with respect to the microbiome. We tested the effect of dose and generally found no effect of dose with respect to the abundance (16S rRNA sequencing) or culture (cfu) data within the individual parity-disease groups. In our mind, this finding justified the pooling of data across doses.

In conclusion, we show different patterns of infection for primiparous compared with multiparous cows with metritis. We propose the possibility that the mechanisms of tolerance and resistance may differ for primiparous and multiparous cows. Further investigation into mechanisms of tolerance and resistance that are affected by parity may yield novel methods to reduce the incidence of postpartum metritis in primiparous cows.

## References

[bib1] Bhargavi G., Subbian S. (2024). The causes and consequences of trained immunity in myeloid cells. Front. Immunol..

[bib2] Bicalho M.L.S., Santin T., Rodrigues M.X., Marques C.E., Lima S.F., Bicalho R.C. (2017). Dynamics of the microbiota found in the vaginas of dairy cows during the transition period: Associations with uterine diseases and reproductive outcome. J. Dairy Sci..

[bib3] Bromfield J.J., Santos J.E.P., Block J., Williams R.S., Sheldon I.M. (2015). Physiology and Endocrinology Symposium: Uterine infection: Linking infection and innate immunity with infertility in the high-producing dairy cow. J. Anim. Sci..

[bib4] Bromfield J.J., Watt M.M., Iacovides S.M. (2018). Characterisation of peripheral blood mononuclear cell populations in periparturient dairy cows that develop metritis. Vet. Immunol. Immunopathol..

[bib5] Bruun J., Ersb⊘ll A.K., Alban L. (2002). Risk factors for metritis in Danish dairy cows. Prev. Vet. Med..

[bib6] Galvão K.N., Bicalho R.C., Jeon S.J. (2019). Symposium review: The uterine microbiome associated with the development of uterine disease in dairy cows. J. Dairy Sci..

[bib7] George D., Mallery P. (2010).

[bib8] Hornef M.W., Wick M.J., Rhen M., Normark S. (2002). Bacterial strategies for overcoming host innate and adaptive immune responses. Nat. Immunol..

[bib9] Laidlaw B.J., Craft J.E., Kaech S.M. (2016). The multifaceted role of CD4(+) T cells in CD8(+) T cell memory. Nat. Rev. Immunol..

[bib10] Lean I.J., Golder H.M., LeBlanc S.J., Duffield T., Santos J.E.P. (2023). Increased parity is negatively associated with survival and reproduction in different production systems. J. Dairy Sci..

[bib11] Lean I.J., LeBlanc S.J., Sheedy D.B., Duffield T., Santos J.E.P., Golder H.M. (2023). Associations of parity with health disorders and blood metabolite concentrations in Holstein cows in different production systems. J. Dairy Sci..

[bib12] LeBlanc S.J. (2023). Review: Postpartum reproductive disease and fertility in dairy cows. Animal.

[bib13] Machado V.S., Bicalho M.L.S., Meira E.B.S., Rossi R., Ribeiro B.L., Lima S., Santos T., Kussler A., Foditsch C., Ganda E.K., Oikonomou G., Cheong S.H., Gilbert R.O., Bicalho R.C. (2014). Subcutaneous immunization with inactivated bacterial components and purified protein of *Escherichia coli, Fusobacterium necrophorum* and *Trueperella**pyogenes* prevents puerperal metritis in Holstein dairy cows. PLoS One.

[bib14] Machado V.S., Silva T.H. (2020). Adaptive immunity in the postpartum uterus: Potential use of vaccines to control metritis. Theriogenology.

[bib15] Moraes J.G.N., Gull T., Ericsson A.C., Poock S.E., Caldeira M.O., Lucy M.C. (2024). The microbiome of the pregnant uterus in Holstein dairy heifers and cows assessed by bacterial culture and 16S ribosomal RNA gene sequencing. Front. Microbiol..

[bib16] Ochando J., Mulder W.J.M., Madsen J.C., Netea M.G., Duivenvoorden R. (2023). Trained immunity — Basic concepts and contributions to immunopathology. Nat. Rev. Nephrol..

[bib17] Sheldon I.M., Cronin J.G., Bromfield J.J. (2019). Tolerance and innate immunity shape the development of postpartum uterine disease and the impact of endometritis in dairy cattle. Annu. Rev. Anim. Biosci..

[bib18] Sheldon I.M., Lewis G.S., LeBlanc S., Gilbert R.O. (2006). Defining postpartum uterine disease in cattle. Theriogenology.

[bib19] Sheldon I.M., Molinari P.C.C., Ormsby T.J.R., Bromfield J.J. (2020). Preventing postpartum uterine disease in dairy cattle depends on avoiding, tolerating and resisting pathogenic bacteria. Theriogenology.

[bib20] Silva J.C.C., Bringhenti L., Siqueira L.C., Rodrigues M.X., Zinicola M., Pomeroy B., Bicalho R.C. (2023). Testing the induction of metritis in healthy postpartum primiparous cows challenged with a cocktail of bacteria. Animals (Basel).

[bib21] Silva J.C.C., Siqueira L.C., Rodrigues M.X., Zinicola M., Wolkmer P., Pomeroy B., Bicalho R.C. (2023). Intrauterine infusion of a pathogenic bacterial cocktail is associated with the development of clinical metritis in postpartum multiparous Holstein cows. J. Dairy Sci..

